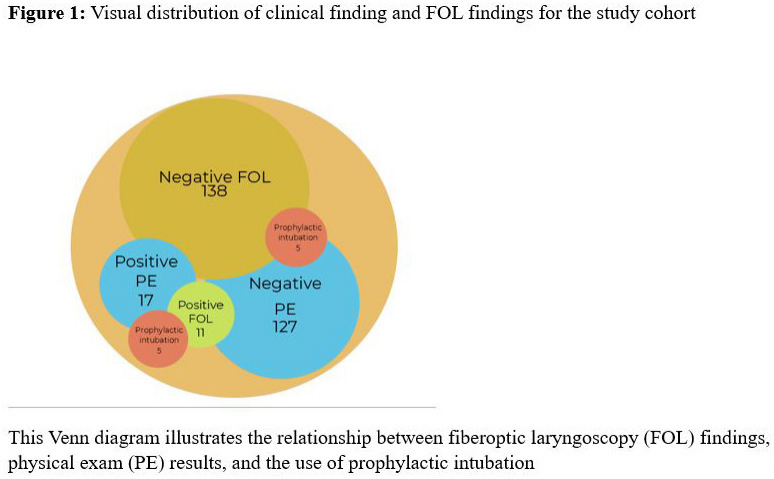# 585 Minimizing Unnecessary Procedures: Revisiting Fiberoptic Laryngoscopy Protocols for Pediatric Burn Patients in the Emergency Department

**DOI:** 10.1093/jbcr/iraf019.214

**Published:** 2025-04-01

**Authors:** Dmitry Kotovich, Stav Sarna Cahan, Lea Ohana Sarna Cahan, Miriam Ben Hamo, Saar Hashavya, Menahem Gross

**Affiliations:** Department of Plastic and Reconstructive Surgery, Hadassah-Hebrew University Medical Center; Hadassah Medical Center; Hadassah Medical Center; Tel-Aviv University; Hadassah Medical Center; Hadassah Medical Center

## Abstract

**Introduction:**

Facial burn injuries in pediatric patients can significantly threaten airway patency, requiring immediate evaluation. Current assessment algorithms mainly rely on clinical judgment, with limited data supporting the use of Fiber Optic Laryngoscopy (FOL) as a supplementary test. This study aims to assess whether FOL is necessary in the routine evaluation of pediatric facial burn patients and recommends its use only when clinical signs of inhalation injury are present.

**Methods:**

This retrospective analysis covered data from all patients aged 0-18 admitted to a Level 1 trauma and burn center with facial burns, who underwent FOL by an ENT physician between January 2010 to December 2022. Data collected included demographics, mechanism of injury, clinical findings, FOL findings, intubation performed, pediatric intensive care unit (PICU) admission and mortality rate. Statistical analysis was performed using Chi-Square or a Fisher’s exact test in R version 4.3.2 with significance set at p < 0.05.

**Results:**

Over the 12-year period, 149 children with scald or flash facial burns underwent documented FOL. The mean age was 1.8 years, with male predominance (68.4%). Scald burns were more common in toddlers (mean age 2.16 years, 69%), while flash burns were prevalent in older children (mean age 10.42 years, 30.8%). Of the patients, 27% with scald burns and 22% with flash burns were admitted to the PICU. Inhalation injury was confirmed in 11.4% of patients. FOL findings indicating inhalation injury were present in 11 (7.3%) of all patients. Of these, 5 (45.5%) had clinical signs of inhalation injury, while 6 (54.5%) had normal exams. FOL had a sensitivity of 29% and specificity of 95% compared to clinical findings. Intubation was performed in 10 (6.7%) of patients, with half showing positive FOL and clinical findings. Twelve children with clinical signs but negative FOL did not require airway protection, and six patients with positive FOL but no clinical signs did not require intubation.

**Conclusions:**

This study demonstrates that Flexible Fiber Optic Laryngoscopy (FOL) has low sensitivity for detecting inhalation injury based on clinical findings. Given the associated pain and discomfort, FOL should not be routinely used in emergency airway assessments for pediatric facial burns and should be reserved for cases with clinical signs suggestive of inhalation injury.

**Applicability of Research to Practice:**

Fiber Optic Laryngoscopy (FOL) should be reserved for pediatric burn patients presenting with clear clinical signs of inhalation injury. This targeted approach minimizes unnecessary interventions and reduces patient discomfort, aligning with best practices for improving outcomes in the pediatric population.

**Funding for the Study:**

N/A